# Validation of the radiological detection of the chorda-facial angle: impact on the round window accessibility during pediatric cochlear implantation

**DOI:** 10.1007/s00330-022-08953-7

**Published:** 2022-06-23

**Authors:** Saad Elzayat, Haitham H. Elfarargy, Rasha Lotfy, Islam Soltan, Hisham N. Lasheen, Valerio Margani, Edoardo Covelli, Maurizio Barbara, Mahmoud Mandour

**Affiliations:** 1 Otolaryngology Department, Kafrelsheikh University, Kafr el-Sheikh, Egypt; 2 Radiology Department, Tanta University, Tanta, Egypt; 3 Otolaryngology Department, Cairo University, Cairo, Egypt; 4 Otolaryngology Department, Sapienza University of Rome, Rome, Italy; 5 Otolaryngology Department, Tanta University, Tanta, Egypt

**Keywords:** Round window, Ear, Computed tomography, Chorda tympani nerve, Facial nerve, cochlear implantation, Chorda-facial angle

## Abstract

**Objectives:**

The facial recess, an essential landmark for the posterior tympanotomy approach, is limited by the facial nerve and the chorda tympani, with a complicated relationship. This study tried to find the most appropriate radiological method to evaluate the chorda-facial angle (CFA). We also checked the effect of this angle on the round window accessibility during cochlear implantation.

**Methods:**

It was a retrospective study that included cochlear implant surgeries of 237 pediatric patients, from September 2016 to April 2021. Two physicians evaluated the CFA in the para-sagittal cut of the preoperative HRCT. The round window accessibility was assessed in the unedited surgery videos.

**Results:**

The CFA ranged from 21° to 35° with a mean of 27.14 ± 3.5°. It was detected in all cases with a high agreement between the two CT reviewers’ measurements. The CFA differed significantly between the accessible group and the group with difficult accessibility (*p* value < 0.001). Spearman’s correlation coefficient revealed a strong correlation between the CFA and the intraoperative round accessibility. 25.5° was the best cutoff point; below this angle, difficult accessibility into the RW was expected, with high sensitivity, specificity, and accuracy

**Conclusions:**

Our study on a relatively large number of cases provided a precise, valid, reliable, and applicable method to evaluate the CFA in the HRCT scan. We found a significant-close relation between the CFA and the round window accessibility; the difficulty increased with a need for posterior tympanotomy modification when the angle decreased.

**Key Points:**

*• Radiological detection of the chorda-facial angle was always problematic, without a previous straightforward method in the literature.*

*• We used the para-sagittal cut of the high-resolution CT scans to evaluate the CFA. This cut was beneficial to seeing the chorda tympani nerve in every examined case. There was a high agreement between the two CT reviewers’ measurements.*

*• Preoperative evaluation of the CFA in the HRCT accurately predicted the round window accessibility. Patients with CFA less than 25.5*^°^ *were expected to have difficult accessibility into the round window during cochlear implantation.*

## Introduction

The chorda tympani nerve (CTN) arises from the mastoid part of the facial nerve (MPVN), then passes anterosuperior in the posterior canaliculus to enter the middle ear [1]. Both nerves (CTN and MPFN) with the incus buttress bound the facial recess. During cochlear implantation (CI), facial recess (FR) is an essential bony landmark for the posterior tympanotomy approach. An accessible round window (RW) is the main target of the preformed posterior tympanotomy for safe atraumatic electrode insertion between the CTN laterally and the MPFN medially [2].

Although the posterior tympanotomy approach during CI is the most common, safe, and practical approach, it still has possible complications. These complications are mostly related to the MPFN and CTN, especially in cases with anatomic variations such as anterior displacement of the MPFN, lateral displacement of the facial nerve, and high origin of the CTN. These variations may increase the difficulty of the accessibility to the RW which is the main target of an efficient electrode insertion into the Scala tympani of the cochlea [3].

HRCT has become the gold standard evaluation before any CI operation. It can detect most of the anatomical structures in the temporal bone with high sensitivity and resolution. So, it helps to see the anatomical variations to predict any possible intraoperative difficulty. This will improve the outcomes with minor consequences [4, 5].

The relation between the CTN and MPFN is complicated. The CTN is lateral to the MPFN in the origin area; then, it becomes anterior. Additionally, the thickness of the CTN is 0.8 mm. These factors make the accurate radiological detection of the CTN and its relation to the MPFN difficult and sometimes impossible. It is problematic to choose the best cut of the HRCT to see the CTN [6, 7].

Many previous studies tried to assess the chorda-facial angle (CFA), the angle between the CTN and the MPFN. Most of these analytic studies were anatomical on cadavers. On the other side, the radiological studies about the CFA are very few and without a straightforward reproducible radiological method to detect it. Also, these studies did not evaluate the impact of the CFA on RW accessibility during CI.

This study proposed a valid simple radiological maneuver to detect the CFA in the HRCT. Also, we assessed the impact of the CFA on RW accessibility during cochlear implantation.

## Materials and methods

### Ethics

We initially obtained the Institutional Review Board approval to conduct this study. The included patients’ guardians signed an informed agreement on using their children’s data in our research.

### Study design

It was a retrospective observational case-series study.

### Setting and duration

Senior CI surgeons (M. Mandour and S. Elzayat) performed the cochlear implant surgeries at tertiary referral institutions of cochlear implantation through the national cochlear implant program from September 2016 to April 2021.

### Subjects

We included 237 pediatric patients who underwent CI surgery through the posterior tympanotomy (PT) approach. We only included patients who had the preoperative HRCT scans, and their unedited surgical video record was present. We excluded cases with previous ear surgeries, middle ear inflammation (cholesteatoma, otitis media with effusion), other approaches for CI, congenital cochlea-vestibular anomalies, external auditory canal (EAC) anomalies, preoperative facial paralysis, and revision CI. We also excluded cases with the radiological length between the chorda tympani nerve origin to the stylomastoid foramen being more than 5.5 mm to avoid the effect of the site of the origin of the CTN on the RW accessibility and focus only on the effect of the CFA. So, we excluded 63 patients of a total of 300 patients to get our study sample (237 patients).

### CT protocol

Radiological images were performed using multi-slice HRCT devices (GE medical systems-128 slices). The lateral scout (55°) was used, starting below the mastoid until obtaining a clear petrous bone. The CT scan data were acquired with the following:

- 0.5-s rotation time
- 0.725 pitch factor
- 140-kV tube voltage
- 125-mA tube current
- 240-mm scan field of view (FOV)
- 16 × 0.5-mm row slice thickness
- 0.5/0.3-mm reconstructed slice thickness/slice interval
- 4500/500 window width/window level

**Fig. 1:**
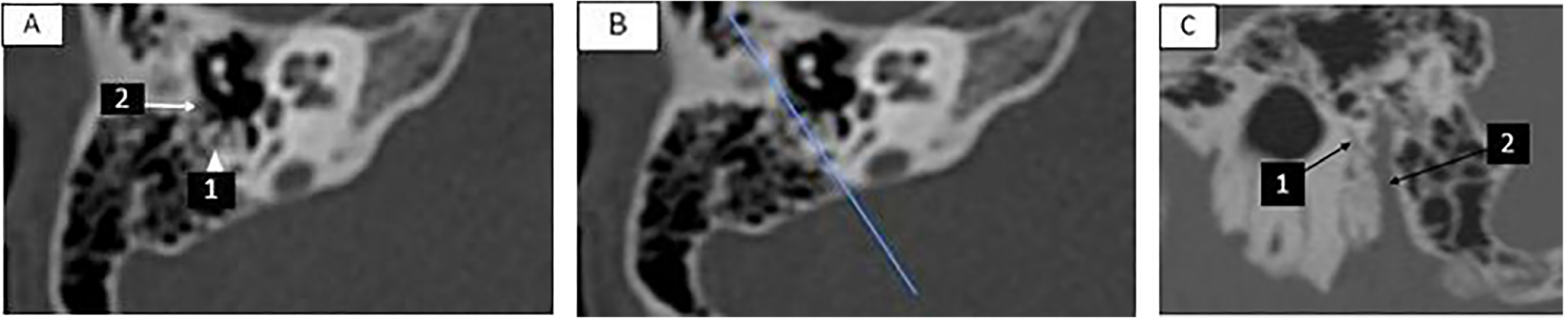
The radiological maneuver of obtaining the chorda-facial angle; **A** right axial cut of the HRCT. (1 = mastoid portion of the facial nerve, 2 = chorda tympani nerve). **B** Blue line joining the mastoid portion of the facial nerve and the chorda tympani nerve; representing the plane of reconstruction for transverse oblique short axis (parasagittal) view. **C** Oblique transverse oblique cut at the plane of the MPFN-CTN line. (1 = chorda tympani nerve, 2 = mastoid portion of the facial nerve)

Post-processing of the scan was done using 0.5-mm raw data that was brought up on the console viewer in three orthogonal planes: axial, coronal, and sagittal. The radiologist scrolled through the sagittal plane until the view of the lateral semicircular canal was obtained represented by the two dots of the anterior and posterior limbs. The axial plane was established by connecting the two dots. The radiologist scrolled through the axial data set until an image of both the MPFN and CTN, appearing as two dots, was obtained. Then, the oblique coronal short-axis view (oblique parasagittal) was reconstructed at a plane parallel to a line joining these two dots representing MPFN+CTN. Fine adjustment of the oblique cut was made until the best visualization of both MPFN and CTN was obtained. Two lines, representing the MPFN and CTN, were inserted. The angle between both lines was measured, representing the CFA (Fig. 1).

### CT reviewing

**Fig. 2:**
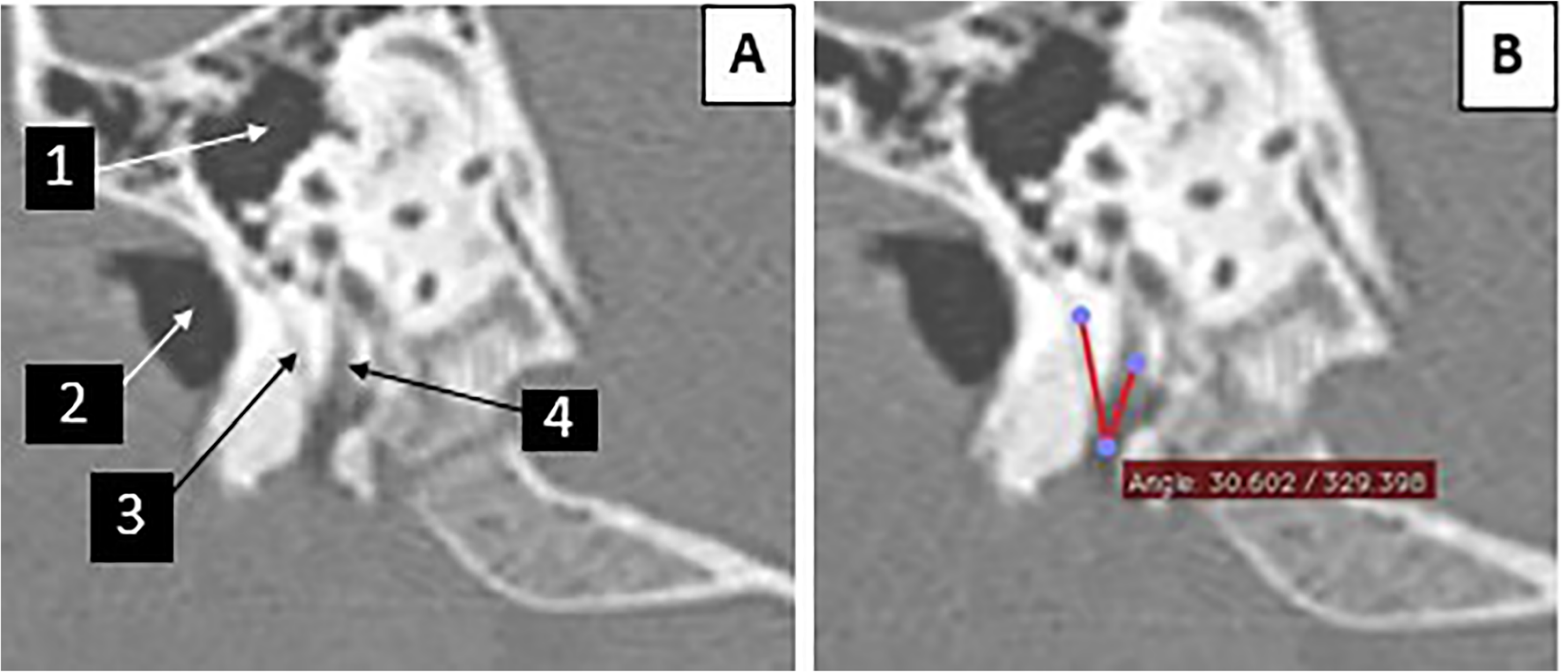
The image of case number 64 (female patient aged 3.8 years). **A** Parasagittal view of the temporal bone. (1 = the middle ear, 2 = the external auditory canal, 3 = the chorda tympani nerve, 4 = the mastoid portion of the facial nerve). **B** The chorda-facial angle = 30.6°

**Fig. 3:**
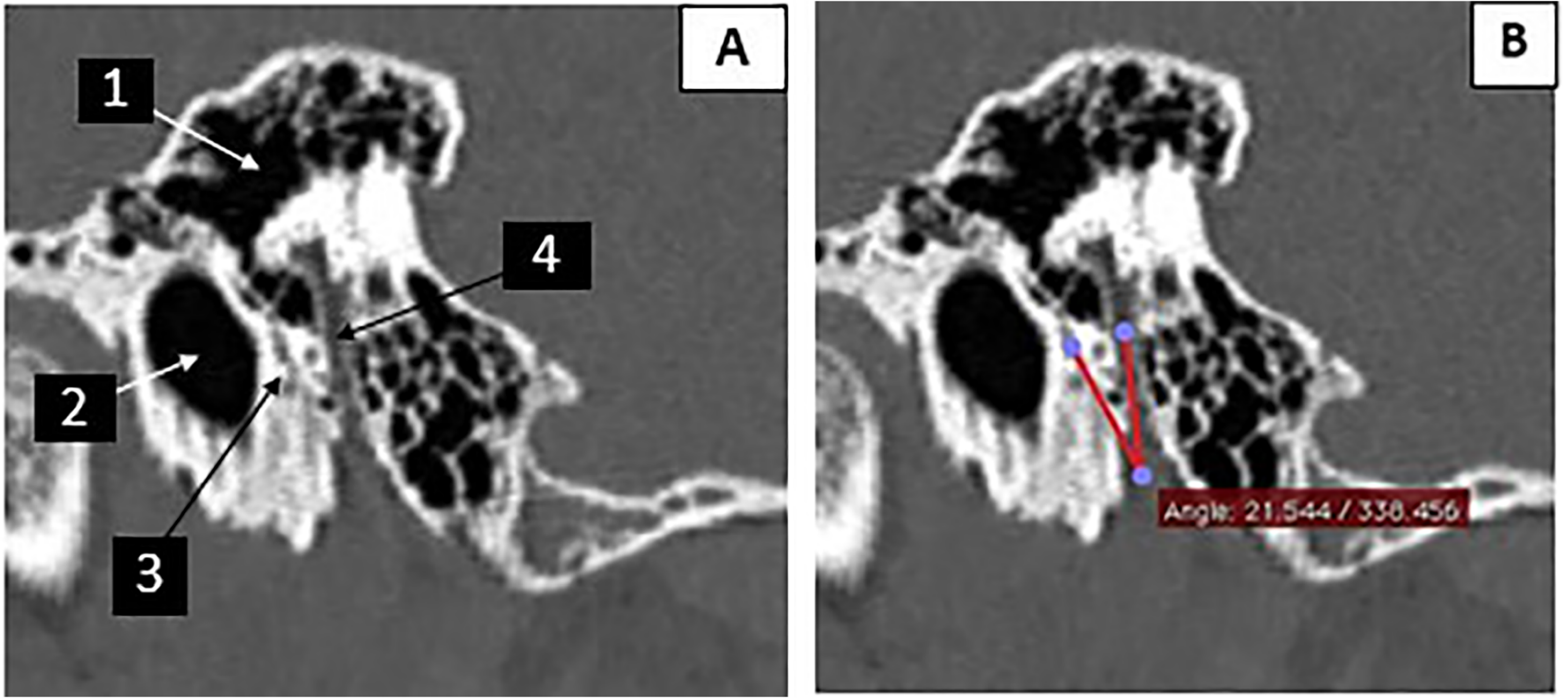
The image of case number 233 (male patient aged 3.2 years). **A** The parasagittal view of the preoperative HRCT. (1 = the middle ear, 2 = the external auditory canal, 3 = the chorda tympani nerve, 4 = the mastoid portion of the facial nerve). **B** the chorda-facial angle = 21.5°

A senior temporal bone radiologist and a CI surgeon reviewed the preoperative HRCT images. They independently measured the CFA in the oblique para-sagittal view of each patient’s HRCT (Figs. 2 and 3).

### Video reviewing

Two CI surgeons, blind to the before-mentioned radiological assessments, independently reviewed the patients’ unedited video records. They judged the RW accessibility through the classic posterior tympanotomy approach (before any need for extra surgical steps). They classified the RW into an accessible RW and RW with difficult accessibility. In cases with difficult access to the RW, the surgeon had to modify the posterior tympanotomy approach to improve the RW accessibility by adding extra surgical steps.

### Outcome measures

We correlated the preoperative radiological measurements of the CFA with the intraoperative RW accessibility classification to evaluate their possible relationship.

### Statistical analysis

Statistical analysis was done using SPSS v22 (IBM© Inc.). Numerical variables were presented as mean and standard deviation (SD). Categorical variables were presented as frequency and percentage (%). We used Mann-Whitney to compare both groups. *p* value < 0.05 was considered significant. We used Spearman’s correlation coefficient to detect the relationship between the intraoperative RW accessibility classification and other variables. There was a significant relationship if the *p* value was < 0.01. We used the intra-class correlation coefficient test to assess the inter-observer variability. We held the receiver operating characteristic (ROC) curve between the radiological CFA measurements and intraoperative RW accessibility classification.

## Results

The patients’ age ranged from 2.1 years to 7.6 years, with a mean of 4.704 ± 1.25 years. We included 137 (57.8%) males and 100 (42.2%) females. Two hundred five (86.5%) patients were implanted on the right side. In contrast, cochlear implantation was done on the left side in thirty-two (13.5%) patients (Table 1). The CT reviewers could measure the CFA in all patients (100%). The angle ranged from 21° to 35° with a mean of 27.14 ± 3.5°. The intra-class correlation coefficient revealed a vital harmony between CT reviewers’ measurements (Table 1).

**Table 1:** Baseline characteristics, radiological and surgical findings of all patients. (*SD* standard deviation, *CFA* chorda-facial angle, *RW* round window, *PT* posterior tympanotomy, *FN* facial nerve, *ICC* intra-class correlation coefficient)

Age	Minimum (years)	2.1
	Maximum (years)	7.6
	Mean ± SD (years)	4.704 ± 1.251
Sex	Males	137 (57.8%)
	Females	100 (42.2%)
Side of operated ear	Right	205 (86.5%)
	Left	32 (13.5%)
Radiological CFA	Minimum (degrees)	21
	Maximum (degrees)	35
	Mean ± SD (degrees)	27.14 ± 3.5
	ICC	0.977
Intraoperative RW accessibility	Accessible	159 (67.1%)
	Inaccessible	78 (32.9%)
	ICC	0.949
Type of PT	Classical PT	159 (67.1%)
	Modified PT	78 (32.9%)
Extra-surgical steps to make the RW accessible	No	159 (67.1%)
	Reallocation of microscope	8 (3.4%)
	Thinning of the meatal wall	13 (5.5%)
	uncapping of the FN	16 (6.8%)
	Sacrificing chorda tympani nerve	11 (4.6%)
	Removal of incus buttress	5 (2.1%)
	Use of endoscope	25 (10.5%)

Patients were divided into two groups according to the intraoperative round window accessibility through the classic posterior tympanotomy approach. The first group (group A) included 159 (67.1%) cases with an accessible RW. On the other hand, group B had 78 (32.9%) cases with difficult access to the RW (Table 2). The observations of both video reviewers were strongly correlated as the intra-class correlation coefficient was 0.949. Both groups did not show statistically significant differences regarding the age, sex, or operation side (*p* value was more than 0.05) (Table 2). The radiological CF differed significantly between both groups as the *p* value was < 0.001 (Table 2).

**Table 2:** Comparison between the accessible RW group and the group with difficult access to the RW. (*SD* standard deviation, *CFA* chorda-facial angle, *RW* round window)

Number		Group A (accessible RW)	Group B (difficult accessibility into the RW)	*p* value
		159 (67.1%)	78 (32.9%)	
Age	Minimum (years)	2.1	2.1	0.87
	Maximum (years)	7.6	7.3	
	Mean ± SD (years)	4.72 ± 1.244	4.65 ± 1.27	
Sex	Males	93 (56.4%)	44 (56.4%)	0.76
	Females	66 (41.5%)	34 (43.6%)	
Side of operated ear	Right	141 (88.7%)	64 (82.1%)	0.16
	Left	18 (11.3%)	14 (17.9%)	
Radiological CFA	Minimum (degrees)	23	21	< 0.001*
	Maximum (degrees)	35	26	
	Mean ± SD (degrees)	29.11 ± 2.45	23.14 ± 1.016	

*Significance as the *p* value was < 0.05

In group B, the posterior tympanotomy approach necessitated extra surgical steps to get an accessible RW. These steps included the reallocation of the microscope head in eight cases, extreme thinning of the posterior meatal wall in thirteen patients, uncapping the mastoid portion of the facial nerve in sixteen subjects, sacrificing the chorda tympani nerve in eleven cases, removal of the incus buttress in five patients, and the use of oto-endoscope in twenty-five cases (Table 1).

Spearman’s correlation coefficient revealed a significant relationship between the preoperative radiological CF angle and the intraoperative RW accessibility as the *p* value was < 0.001. The negativity of Spearman’s correlation coefficient indicated an inverse relationship between the CFA and the RW accessibility; when the angle decreased, the difficulty increased and the reverse. In contrast, the RW accessibility and the radiological CFA did not show statistical significance correlations with the age, sex, or the operation side as the *p* values were > 0.01 (Table 3).

**Table 3:** Correlation of the RW accessibility and the radiological CFA with other variables

	RW accessibility		Radiological CFA	
	Spearman correlation coefficient	*p* value	Spearman correlation coefficient	*p* value
Age	0.01	0.87	0.042	0.52
Sex	0.02	0.76	0.024	0.71
Operation side	0.091	0.16	0.063	0.34
Radiological CFA	− 0.807	< 0.001*		

^*^Significance as the *p* value was < 0.01

ROC confirmed the close relationship between the preoperative radiological CFA and the intraoperative RW accessibility, as the area under the curve was 0.993. ROC curve also revealed that the best cutoff point was 25.5. This angle had a high sensitivity (98.72%), specificity (97.48%), and accuracy (97.89%) in the preoperative radiological prediction of RW accessibility. This angle’s positive predictive value was 95.06%, the negative predictive value was 99.36%, and the Youden index was 0.96.

## Discussion

An accessible round window is the main target of the posterior tympanotomy approach during cochlear implantation for safe atraumatic electrode insertion. MPFN and CTN are among the main limitations of an efficient PT. Furthermore, they may be injured during this common technique with unfavorable consequences such as taste loss and facial paralysis. The preoperative HRCT could help detect these two nerves’ anatomic variations. This would improve the surgical outcomes [8].

It is usually problematic to assess the complicated relationship between the CTN and the MPFN. Besides the very narrow CTN canal (0.8 mm), the origin of CTN is lateral to the MPFN; then, it passes into the posterior canaliculus anterior to the MPFN. This relationship is usually indiscernible in the conventional cuts (coronal, axial, and sagittal) of the HRCT. Bettman et al’s radiological analysis of the FR dimensions could not detect the CTN and depend on the distance between the MPFN and the annulus during the evaluation of FR width [9]. On the other hand, many studies measured the FR width in the axial cut of the HRCT [10]. The CTN is usually tiny in this cut and may be mistaken as an air cell. FR width may assess the oval or round window areas [11]. So, the use of FR width, the distance between the anterolateral surface of the MPFN and the CTN or the annulus in the axial cut of the HRCT, is inaccurate. These variations would make the CFA preferable and reliable to evaluate the FR in the HRCT, as it is more related to the round window area.

CFA was an exciting point for many of the previous anatomical research. On the other hand, only a few radiological trials evaluated the CFA. The CFA was 24.8° in the study of Zhu et al on forty ears of twenty cadavers [12]. Calli et al measured the CFA in their research on thirty cadavers, and it was 23.58 ± 6.84° [13]. According to the study of Măru et al on thirty-five cadavers, the CFA ranged from 26 to 35° [14]. Additionally, the CFA ranged from 25 to 28.69° in the study of Jain et al on thirty-five cadavers [15].

Diogo et al used cone beam CT for the radiological evaluation of the CFA in eighty-two patients. They detected the angle only in sixty-two (75%) patients. It was 22.6 ± 9.5°. They did not mention the radiological cut in which he measured the CFA [16]. The radiological study of Jeon et al on twenty adult patients used a complicated 3-D reconstruction technique to evaluate the CFA, which was 18.4 ± 1.05° [17]. McManus et al used a micro-CT scanner to assess the angle between the posterior canaliculus (containing the CTN) and the FN on forty cadavers. He found the CFA was 18 ± 9° in the sagittal plane and was 13 ± 6° in the coronal plane [18]. These anatomical and radiological studies did not correlate with the CFA and intraoperative findings.

Our study used the oblique para-coronal long axis (para-sagittal) cut of the HRCT to detect the CFA. This view has become a routine before CI surgeries as it can efficiently evaluate the FN [19]. Scrolling through the cuts with this obliquity helped to see the CFA in every included case. This indicated the validation of this method to detect the CFA in the HRCT. The reliability of this method was also confirmed by the high agreements between the two measurements of the CT reviewers. We tried to provide the radiologists and CI surgeons with an accurate, applicable way to measure the CFA in the preoperative HRCT scan.

The CFA had a potent and significant correlation with intraoperative RW accessibility. When the angle decreased, the difficulty increased. In these cases, more surgical steps may be needed to modify the posterior tympanotomy approach to get an accessible RW. This correlation was the first to assess the relationship between the CFA and RW accessibility.

According to our study, cases with CFA less than 25.5° may have an intraoperative difficult accessibility into the RW. This cutoff angle’s sensitivity, specificity, and accuracy were very high. This could help the surgeon in the preoperative difficulty prediction with an efficient preparation of the CI surgery.

### Benefits of our study

Our study had two main benefits. The first benefit was to provide the radiologists with an easy and applicable maneuver to measure the CFA accurately. The second benefit was to beforehand prediction of the RW accessibility. By this prediction, the surgeon will be ready for the possibility of performing extra-surgical steps with the availability of other tools such as an oto-endoscope and extra tiny drilling burrs. He may prepare bone pate to close any defect in the posterior wall of the EAC if its extreme thinning would be needed in problematic cases. The complex operation may be referred to another more skillful senior surgeon. Furthermore, another approach may be used from the start in cases of predicted difficult accessibility to the RW. We aimed to improve the quality of life of the CI candidates by avoiding any unnecessary hazards and improving the outcomes. For example, in cases with difficult accessibility into the RW due to narrow CFA, the surgeon may have to sacrifice the CTN with consequent loss of taste sensation. This would impact badly the chefs’ quality of life. In this situation, another approach would be preferred from the start.

## Conclusions

Our study on a relatively large number of cases provided a precise, valid, reliable, and applicable method to evaluate the CFA in the HRCT scan. CFA was detected clearly in all cases in the parasagittal cut. We were the first to assess the relationship between the preoperative CFA and the intraoperative RW accessibility. We found a significant-close relation between them; the difficulty increased with a need for posterior tympanotomy modification when the angle decreased. CFA with 25.5° was the best cutoff angle of prediction the RW accessibility with high sensitivity, specificity, and accuracy.

## Abbreviations

AUC: Area under the curve
CFA: Chorda-facial angle
CI: Cochlear implantation
CTN: Chorda tympani nerve
FN: Facial nerve
FR: Facial recess
HRCT: High-resolution computed tomography
MPFN: Mastoid portion of the facial nerve
PT: Posterior tympanotomy
ROC: Receiver operating characteristic curve
RW: Round window

## Declarations

### Guarantor

The scientific guarantor of this publication is professor Maurizio Barbara:

Director of NESMOS Department

PhD Coordinator in “Sensorineural Plasticity”

Director UOC ORL Az. Osp-Universitaria Sant’Andrea

Medicine and Psychology

Sapienza Rome

maurizio.barbara@uniroma1.it

### Statistics and biometry

The author (Haitham H. Elfarargy) has significant statistical expertise.

### Methodology

• retrospective

• observational

• multicenter study
